# Supplementary figures and images for: Molecular mechanism of point mutation-induced Monopolar spindle 1 (Mps1/TTK) inhibitor resistance revealed by a comprehensive molecular modeling study
Source: PeerJ. 2019 Jan 21;7:e6299. doi: 10.7717/peerj.6299 (PMC6345219; doi:10.7717/peerj.6299)

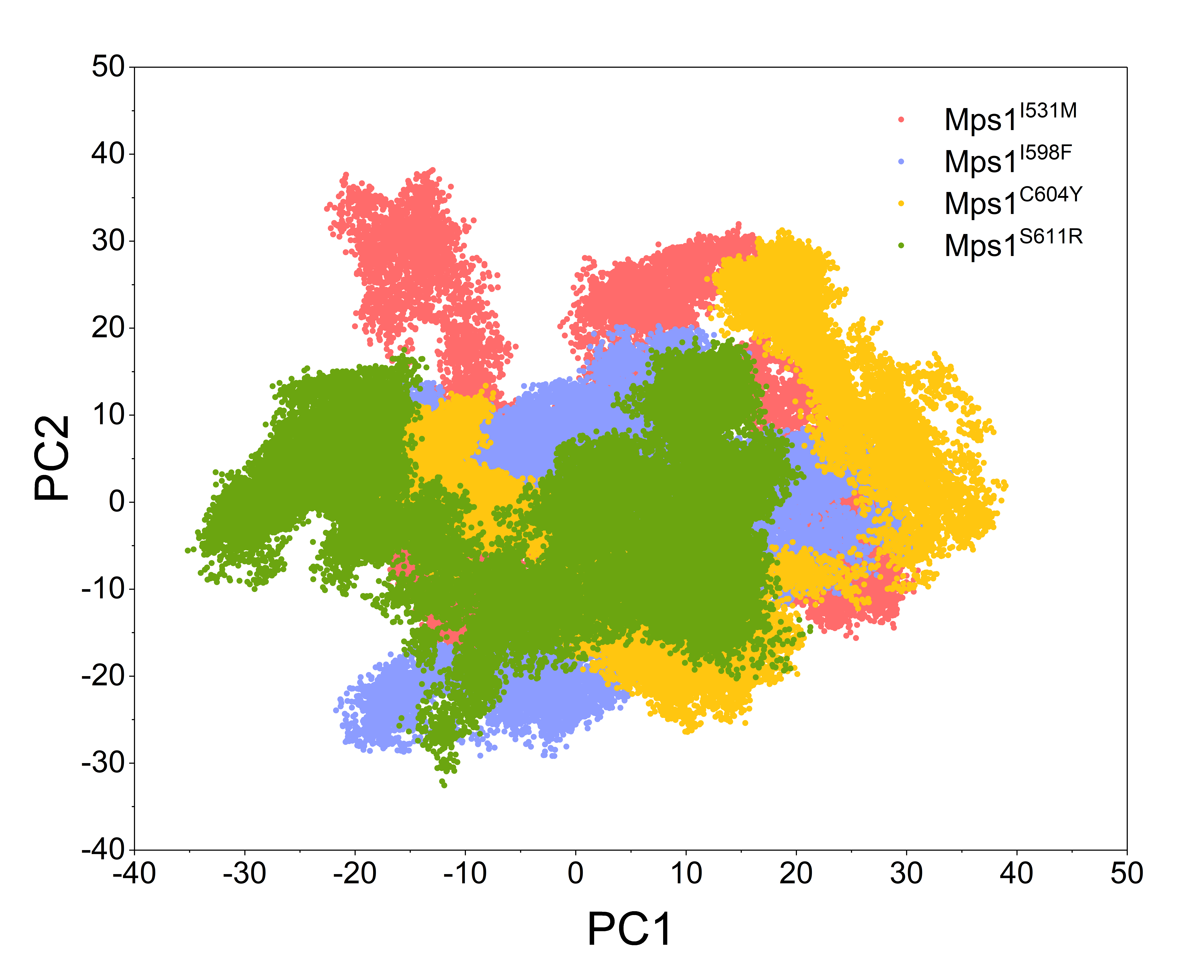

Supplement: Supplemental Information 1 [file peerj-07-6299-s002.png]
